# Response of Maize (*Zea mays* L.) to Drought under Salinity and Boron Stress in the Atacama Desert

**DOI:** 10.3390/plants12071519

**Published:** 2023-03-31

**Authors:** Camilo Riveros-Burgos, Richard Bustos-Peña, Wladimir Esteban-Condori, Elizabeth Bastías

**Affiliations:** 1 Institute of Agri-Food, Animal and Environmental Sciences (ICA3), Universidad de O’Higgins, San Fernando 3070000, Chile; 2 Departamento de Producción Agrícola, Facultad de Ciencias Agronómicas, Universidad de Tarapacá, Casilla 6-D, Arica 1000000, Chile

**Keywords:** water potential, leaf gas exchange, grain yield, Lluta valley

## Abstract

The Lluta valley in northern Chile is a hyper-arid region with annual precipitation lower than 1.1 mm, and high levels of boron (B) from alluvial deposits are present together with other salts that originated from the Cretaceous. Under these abiotic conditions, the ‘lluteño’ maize (*Zea mays* L.) is of interest because it has adapted to the Lluta valley with high salinity levels and B excess in the soil and irrigation water. Water and salt stress coincide in heavily irrigated hyper-arid agricultural areas, yet they are usually studied in isolation. We investigated in field conditions the combined effects of drought (22 days with no irrigation) under salinity (ECe: 5.5 mS cm^−1^; Na+: 17.8 meq L^−1^) and B (21.1 meq L^−1^) stress on physiology, growth, yield, and hourly water relations. The results allow to hypothesize that the measurement of the pre-dawn water potential represents the balance between the water potential of the soil and the root. Besides, under drought a significant effect of irrigation and time interaction was observed presenting a high differential between the leaf and stem water potential in both phenological stages. Furthermore, a decrease in net assimilation was observed, and it could be explained in part by non-stomatal factors such as the high radiation and temperature observed at the end of the season. Despite the drought, the cobs did not present a significantly lower quality compared to the cobs of plants without stress.

## 1. Introduction

It is well known that changes in environmental conditions could adversely impact agricultural productivity [1]. The current climatic condition leads to a temperature rise and is likely to add 1.8 to 4 °C to present-day temperature by the turn of this century. In this regard, a consequence of climate change is the expansion of arid and semi-arid regions, where usually several crops are grown under excessive concentrations of soluble mineral elements in both the soil and irrigation water [2,3]. In this environmental condition, a clear problem is the salinity and boron-induced toxicity, which has been described in different geographical locations [1,4]. Individually, boron (B) toxicity and salinity stress are well recognized as severe stress conditions for plants. Moreover, these stress conditions not only occur individually but also co-exist in alkaline soils in low rainfall regions, such as northern Chile [5]. Hence, according to Naz et al. [6] salinity stress seems to alter B toxicity symptoms by developing water stress in plants and consequently increasing the soluble B concentrations in different spaces, thus evidencing even more the symptoms of B toxicity.

Maize (*Zea mays* L.) is a cereal crop, which is grown worldwide [7]. It is a domesticated crop of the Americas, and its presence in South America has been estimated between 4500 years BP [8,9]. The species stands out because of its physiological mechanism (C4 metabolism) and resilience, considering the wide range of environmental conditions where it is grown, including several altitudes and latitudes. Nevertheless, it is necessary to improve the drought resistance for increasing the maize yield under water-limited conditions [10]. For that, it is important to recognize the mechanism of tolerance or response of maize to drought and salinity condition associated with B toxicity. According to Katerji et al. [11], the effect of salinity or drought on maize yield does not present a clear differentiation. For instance, Cakir [12] differentiated that maize yield response depending on the plant development stage. Thus, the maize would be relatively tolerant to water deficits during the vegetative and ripening periods and sensitive to drought during the flowering period. Regarding B, maize is a species sensitive to high concentrations of this element [2]; however, the response of maize to water deficit in conditions of B excess is unknown.

The Lluta valley in northern Chile is a hyper-arid region with annual precipitation lower than 1.1 mm, and high levels of B coming from alluvial deposits are present together with other salts from Cretaceous shales [13]. Due to these environmental factors, the agronomic productivity in the region is limited to a bunch of relatively salt-tolerant ecotypes or local varieties. In this sense, the maize ecotype “lluteño” is adapted to the Lluta valley, where high levels of salinity and B excess in the soil and irrigation water are found [14,15,16]. This sweet maize has shown remarkable tolerance to salinity and high B content due to continuous selection by the farmer in this valley [17]. Previous results indicate the expression pattern of some stress-related genes in “lluteño” maize combined with high salinity and excess B stress because of physiological parameter changes and more severe damage.

Despite the frequent existence of combined salinity and drought stress episodes under field conditions, little is known of the morpho-physiological responses of lluteño maize to combined stresses. The essential physiological response to low water availability is the decrease in plant photosynthesis, which leads to a lower yield [17]. This response is related to a decrease in leaf water potential and stomatal conductance [18,19]. However, scarce information about daily trends of water status is available for maize under field conditions with high-salinity and B-excess to understand how this species regulates the response to water reduction. In this regard and considering the maize sensitivity when a water reduction occurs, the use of regulated deficit irrigation (RDI) looks like an interesting water-saving tool. The RDI controls soil water content during the season, where irrigation is cut off at certain times, and this practice has presented promising results in fruit production, sugar beet, and cotton, where yield was largely maintained and product quality was increased in some cases, while the irrigation was substantially reduced [20]. A previous study showed that the interaction between mild water and salt stress reduced the transfer of nutrients to the reproductive organs and caused adverse effects on the formation of grains, but the difference in yield between a tolerant and sensitive genotype was not significant [21].

Considering the scarce information about maize response to the interaction of drought with high-salinity and B excess under field conditions, this study aims to evaluate the physiological and commercial response of the maize ecotype “lluteño” to regulated deficit irrigation under high levels of salinity and B excess.

## 2. Results

### 2.1. Meteorological and Soil Conditions

During the study (August to December 2019), the observed atmospheric conditions in the different phenological stages of the “lluteño” maize crop (Supplementary Table S1) showed null rainfall, confirming the classic characteristic aspects of a hyper-arid climate. Most sunny days were observed during the measurements with daily values of solar radiation (Rs) between 24.2 and 30.9 MJ m^−2^.

**Figure 1:**
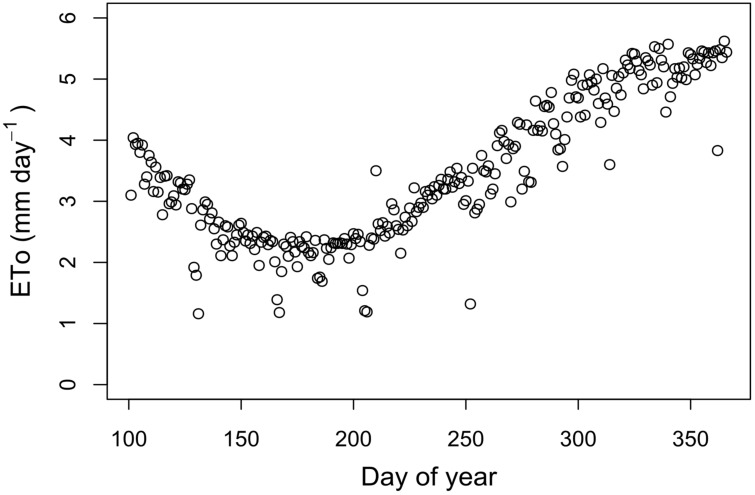
Reference evapotranspiration (ETo) trend of the experimental site during the trial, computed with automatic weather station data and the Penman–Monteith model.

The atmospheric parameters measured in the flowering (VT) stage were hotter and drier than in the vegetative growth (V10), where the average values of air temperature (Ta), vapor pressure deficit (VPD), and wind speed (u) ranged between 19.2–20.0 °C, 0.8–0.9 kPa, and 0.70–0.94 m s^−1^, respectively. Additionally, the reference evapotranspiration (ETo) during the trial evaluations (October and November 2019) presented extreme values of 1.19 and 5.42 mm day^−1^ (Figure 1).

The observed depletion of θ in both treatments was similar until the seventh day after the last irrigation and without essential variations in the different depths of each treatment. The T2 after 11 days after the first irrigation presented the most critical variations concerning the initial values in the V10 and VT stages, with a decrease of 8.5% and 14.5%, respectively (Supplementary Table S2).

### 2.2. Water Status

**Figure 2:**
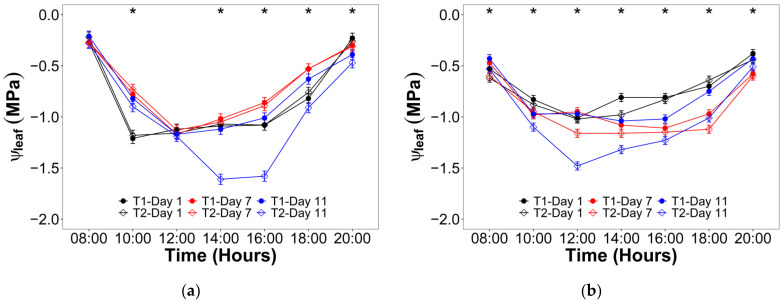
Hourly evolution of leaf water potential (ψ_leaf_) for the irrigation treatments (T1 = Control; T2 = Drought) during the three evaluated days in: (**a**) Vegetative stage; (**b**) Flowering stage. Significant differences between irrigation treatments in the three evaluated days for every hour are indicated by an asterisk.

Respect the leaf water potential (ψ_leaf_), it was observed a significant interaction between the irrigation treatment and both the hourly and daily scale time during V10 and VT (Figure 2). For the V10 stage (Figure 2a), the maximum ψ_leaf_ was observed at 8:00 and 20:00 with values mainly about −0.25 MPa, while the minimum ψ_leaf_ took place between 14:00 and 16:00 for T2 after 11 days with no irrigation and values of −1.61 and −1.57 MPa, respectively. In the VT stage (Figure 2b), the maximum ψ_leaf_ was also observed at 8:00 and 20:00, but with values around −0.50 MPa. On the other hand, the minimum ψ_leaf_ was between 12:00 and 14:00 for T2 after 11 days with no irrigation, reaching values of −1.48 and −1.32 MPa, respectively.

**Figure 3:**
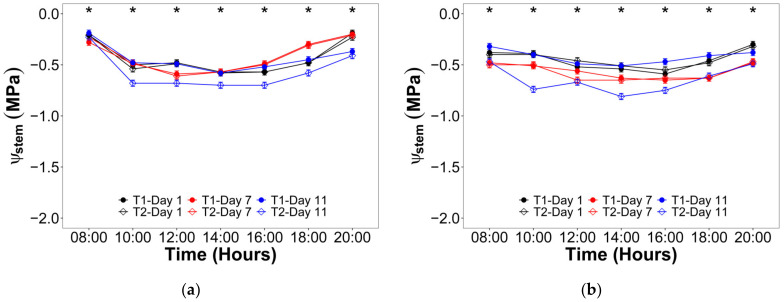
Hourly evolution of stem water potential (ψ_stem_) for the irrigation treatments (T1 = Control; T2 = Drought) during the three evaluated days in: (**a**) Vegetative stage; (**b**) Flowering stage. Significant differences between irrigation treatments in the three evaluated days for every hour are indicated by an asterisk.

For the stem water potential (ψ_stem_), the interaction between the irrigation treatment and both the hourly and daily scale time was significant during both studied phenological stages (Figure 3). The maximum ψ_stem_ values during the V10 stage were recorded at 8:00 and 20:00, rounding the −0.20 MPa (Figure 3a) for both irrigation treatments two and seven days after the trial beginning. Meanwhile, the minimum ψ_stem_ values reached −0.70 MPa between 10:00 and 16:00 for T2 after 11 days without irrigation. For the VT stage (Figure 3b), the maximum ψ_stem_ values were also observed at 8:00 and 20:00, but with a broader range between −0.50 and −0.30 MPa. In this regard, the minimum ψ_stem_ values ranged from −0.81 to −0.67 MPa between 10:00 and 16:00 for T2 after 11 days with no irrigation.

**Figure 4:**
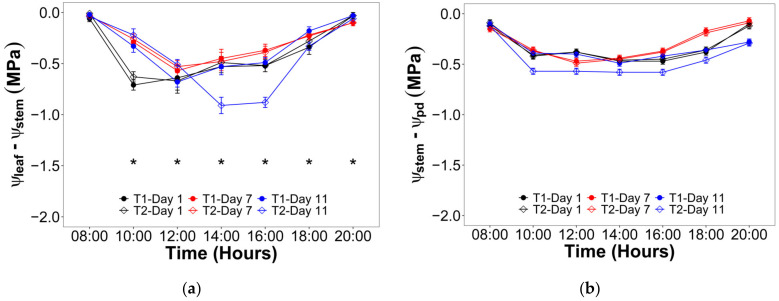
Hourly evolution of the water potential differential during the three evaluated days in the vegetative stage between: (**a**) Leaf and stem (ψ_leaf_ − ψ_stem_); (**b**) Stem and root represented by the predawn water potential (ψ_stem_ − ψ_pd_). Significant differences between irrigation treatments in the three evaluated days for every hour are indicated by an asterisk.

**Figure 5:**
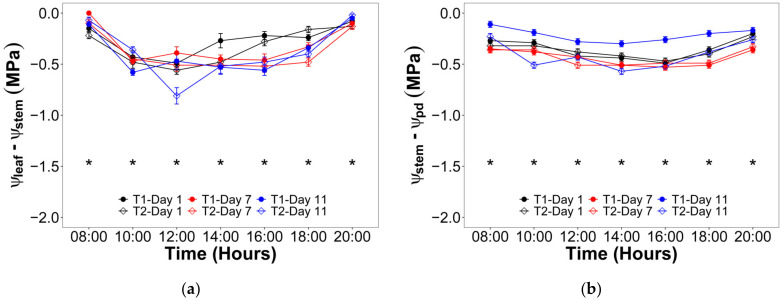
Hourly evolution of the water potential differential during the three evaluated days in the flowering stage between: (**a**) Leaf and stem (ψ_leaf_ − ψ_stem_); (**b**) Stem and root represented by the predawn water potential (ψ_stem_ − ψ_pd_). Significant differences between irrigation treatments in the three evaluated days for every hour are indicated by an asterisk.

For the V10 stage, the hourly evolution of the differential between ψ_leaf_ and ψ_stem_ showed a significant interaction of the irrigation levels and times (hours and days) (Figure 4a). In this way, the lowest differentials were observed at 08:00 and 20:00 near to zero for all treatments and days, while the greatest differential took place between 14:00 and 16:00 for T2 after 11 days with no irrigation with values around −0.90 MPa. On the other hand, the hourly evolution of the differential between ψ_stem_ and predawn water potential (ψ_pd_) did not show a significant interaction of the irrigation levels and times (hours and days) (Figure 4b), and the values ranged between −0.60 and −0.10 MPa. During the VT stage, a similar trend was observed, i.e., there was a significant effect of the interaction between irrigation and the hours and days over the differential between ψ_leaf_ and ψ_stem_ (extreme values of −0.80 and 0.00 MPa) (Figure 5a), while the hourly evolution of the differential between ψ_stem_ and ψ_pd_ was not affected by the irrigation nor the time (extreme values of −0.60 and −0.10 MPa) (Figure 5b).

### 2.3. Leaf Gas Exchange

Table 1 shows a summary of the analysis of variance (ANOVA), for the parameters of gas exchange (A: net assimilation, g_s:_ stomatal conductance, and E: leaf transpiration). It should be noted that the significance values for the analysis of individual factors did not show significant differences. However, when analyzing the factor interaction, significant differences were found for the variable A for both V10 and VT stages, while g_s_ and E were significantly affected only in VT stage. Thus, the irrigation by time interaction showed mean values for A of 32.1 and 41.1 µmol CO_2_ m^−2^ s^−1^ for T1 during days 1 and 11, respectively. Additionally, T2 induced a significant A decrease during VT stage, presenting mean values of 28.8 µmol CO_2_ m^−2^ s^−1^ on day 1 and 20.1 µmol CO_2_ m^−2^ s^−1^ for day 11 after irrigation. For g_s_ and E, a similar trend is observed, showing significant differences in the VT stage, with mean g_s_ values of 0.17 mol H_2_O m^−2^ s^−1^ for T1 for both days (1 and 11) after irrigation, and 0.16 and 0.10 mol H_2_O m^−2^ s^−1^ for T2 in days 1 and 11 after first irrigation. Meanwhile, E presented mean values of 4.11 and 5.05 mmol H_2_O m^−2^ s^−1^ for T1 and 4.23 and 3.40 mmol H_2_O m^−2^ s^−1^ for T2 for both days (1 and 11) after irrigation, respectively.

The data obtained in the study showed that the fresh corn yield was not significantly affected by the water deficit (Table 2), since the mean yield were 5350 and 5337 kg ha^−1^ (relative drop in yield of 0.24%) for the T1 and T2, respectively. However, differences were observed in the accumulation of harvested dry biomass with respect to the total aerial biomass (harvest index). In this sense, the T1 treatment reached a mean harvest index of 20%. Meanwhile, the T2 treatment presented a significantly lower dry biomass (economically marketable product) with a decrease of 8% respect to non-drought stress treatment.

## 3. Discussion

The study of maize physiological response to drought stress under B stress was challenging, considering that literature discussing both topics separately and simultaneously is very scarce. During the trial, the maize plants of the T1 were well irrigated (−0.50 MPa < ψ_leaf_ < −0.25 MPa; −0.60 MPa < ψ_stem_ < −0.20 MPa), while those of the T2 were stressed (−1.67 MPa < ψ_leaf_ < −1.32 MPa; −0.81 MPa < ψ_stem_ < −0.20 MPa). This water status agrees with the reported values found in the literature, which showed that ψ_leaf_ ranged between −1.80 and −0.20 MPa for maize plants grown in pots [22,23,24,25,26] and under field conditions [27,28,29]. In contrast, the ψ_stem_ range has been reported between −0.79 and −0.20 MPa for unstressed plants and between −1.10 and −0.20 MPa for stressed plants [22,28]. 

In this trial, it was hypothesized that measured ψ_pd_ represented the roots and soil water potential equilibrium. These hypothetical conditions allowed obtaining an hourly evolution of hydraulic segmentation. In this regard, when plants are subjected to severe drought, they could lay down more vulnerable and expendable organs (leaves and roots) in favor of stems, according to the hydraulic vulnerability segmentation [30]. While it is true that T2 was not a severe drought, its combined effect with high salt and B content in soil and water could be likened to those conditions. In this way, it can be observed in Figure 4 that there were significant effects of irrigation treatment and time on the T2, presenting the highest water potential differential for ψ_leaf_ − ψ_stem_ during vegetative and flowering stages. Additionally, both figures showed that the differential ψ_stem_ − ψ_pd_ was not affected in any phenological stages. These results suggest that lluteño maize would prioritize increasing the xylem tension avoiding the stomatal closure. However, this response was valid only in the vegetative stage, while the flowering evidenced a lower ψ_leaf_ − ψ_stem_ differential and a significantly lower stomatal conductance (Table 3). Thus, this observed trend suggests that lluteño maize would not risk the production trying to maintain the gas exchange rates like in the vegetative stage.

The response of gas exchange parameters did not present significant differences when analyzing the factors in the vegetative stage (control and stress) and time (day 1 and day 11) [15,31]. However, analyzing the irrigation by time interaction, a decrease in A was observed during the VT stage, varying between 10% (day 1) and 50% (day 11), which agrees with what was proposed by Bhusal et al. [32]. In addition, the same authors proposed this trend for local ecotypes and cultivars, as in lluteño maize. For g_s_ and E, a similar tendency to decrease is shown for the VT stage; these variations are lower, reaching averages of 10% for day 1 and 40% for day 11 in both parameters. This decrease in the gas exchange parameters for the VT stage can be explained in part by the increase in temperature and radiation towards the end of the season (Table 1), which implies thermal stress in the leaf, which coincides with those proposed by Bastías et al. [11], Bhusal et al. [33], and Tiwari and Yadav [34]. However, this condition did not affect the performance, since no significant differences were observed for this parameter.

Despite the moisture deficit in the soil profile in T2 during V10 and VT, the irrigation treatments did not present significant differences in the obtained fresh cobs (kg ha^−1^) and the number of grain rows per ear. These results differ from the literature, mainly in stages defined as sensitive (spike and/or maturation) [12]. For maize hybrids (cv. Xianyu 335) that were irrigated manually for 110 days, Huang et al. [35] observed that water restrictions in seedling, spike, and filling phenological stages generated decreases in grain yield of 47.3% with respect to unstressed crops. Conversely, Oktem et al. [36], in drip-irrigated hybrid maize (cv. Merit), obtained drops of 55% in ear yield for fresh consumption. Likewise, Gomaa et al. [37] reported reductions in most ear quality characteristics with irrigation intervals of 20 days and applications of potassium silicate in hybrid yellow maize (cv. SC168). This null downward trend in yield as irrigation frequency increases is possibly explained by the genetic ability of the species not to show a significant effect on yield.

The studies carried out under field conditions and in the place of origin of the crop guarantee continuous evolution and adaptation to changing climatic conditions, as is the case of “lluteño” maize, a sweet ecotype that is highly tolerant to salinity, excess B, both in the irrigation water and in the soil, and drought stress.

Using local agricultural varieties is an excellent alternative to sustainable agronomic management, where production levels close to modern or hybrid maize can be achieved. This gene pool is invaluable for adapting agricultural systems to future climates. 

## 4. Materials and Methods

### 4.1. Study Site

A study was carried out to evaluate the response of maize (*Zea mays* L. type amylacea ecotype lluteño) to the water deficit during the 2019 growing season. For this purpose, an experimental plot was established in the Lluta valley (18.41° S, 70.12° W, 387 m above sea level), Arica y Parinacota Region, Chile (Supplementary Figure S1). The maize was furrow irrigated, according to the management of Lluta valley growers.

The climate is typically hyper-arid, with infrequent rainfall [38], resulting in an average rainfall of less than 5 mm in recent years [39]. The thermal regime presents a mean annual temperature of 19.1 °C [40]. The alluvial soils of the study field are of the medium to thick family of the Huaylacán series [41], which belongs to the coarse-loamy family over sandy, mixed, thermic Aquollic Salorthids [42]; additionally, there is the presence of salts in the strata of the profile [43,44].

The soils of the Lluta valley are characterized by important limitations for agricultural production due to the low quality of soil and irrigation water (Table 3). The main problem is related to high levels of salinity and B [41], which has generated a low development in local agriculture.

### 4.2. Measurements

#### 4.2.1. Meteorological Measurements

The meteorological variables (air temperature, Ta: relative air humidity, HR: wind speed, u: precipitation, rain: and solar radiation, Rs) were measured within the area of the “lluteño” maize crop. In addition, reference evapotranspiration (ETo) was calculated using the FAO56 Penman–Monteith method [45] using meteorological variables recorded at 30-min intervals from an automatic weather station (WatchDog 2900ET, Spectrum Technologies, IL, USA).

#### 4.2.2. Soil Water Content

θ was measured in the center of the irrigation furrow at two soil depths: 0–20 cm and 20–40 cm. On each sampling date, a gravimetric sample was taken at a single point of each repetition. The gravimetric sample was taken by inserting a 250 cm^3^ aluminum capsule, which was put in the cold to preserve the mass of the sample. The gravimetric samples were weighed and placed in a forced air oven at 105 °C for 24 h in the laboratory. Subsequently, the samples at room temperature were weighed, and from there, the gravimetric humidity (H_g_) was determined, according to Weil and Brady [46]:

H_g_ = M_w_/M_s_, (1)

where H_g_ is the gravimetric water content (g g^−1^), M_w_ the water mass (g), and M_s_ the dry soil mass (g). The bulk density (δ_α_) was determined at the same extraction points of the gravimetric samples. However, the samples were extracted with a stainless steel cylinder of known volume (66.34 cm^3^). The extraction tried to ensure that the sample was flush with the cylinder’s edge without disturbing it. The samples were placed in a forced air oven at 105 °C and weighed at room temperature. From the mentioned variables, the volumetric humidity (θ) was determined, according to Weil and Brady [46], for each monitoring depth:

θ = H_g_*δ_α_, (2)

where θ is the volumetric water content (cm^3^ cm^−3^) and δ_α_ is the bulk density (g cm^−3^).

#### 4.2.3. Plant Water Status

The maize water status was monitored using the three plant water potential methodologies: Predawn (ψ_pd_), leaf (ψ_leaf_), and stem (ψ_stem_), using a pressure chamber (Model 3000F01 Plant Water Status Console, Soil Moisture Equipment Corp., Santa Barbara, CA, EE.UU.) with a grass compression gland sealing system. The potentials were measured in fully expanded healthy leaves and exposed to sunlight. These samples were extracted between the third and fifth pair of leaves from the top of the plant. ψ_pd_ was measured in a total of 12 leaves (a leaf per plant) collected at 5:00 (Coordinated Universal Time UTC—4). While ψ_leaf_ and ψ_stem_ were measured in 168 leaves (84 for ψ_leaf_ and 84 for ψ_stem_) collected every two hours from 8:00 to 20:00 (UTC—4). For the ψ_stem_ measurements, the leaves were wrapped in plastic and covered in aluminum at least 45 min before the measurements [28]. For the three methodologies, once the leaf was extracted, a perpendicular cut to the leaf was made 30 cm from the tip, and a 2 to 3 cm wide leaf blade was left outside the chamber to observe the meniscus [47].

#### 4.2.4. Leaf Gas Exchange

Net assimilation (A), stomatal conductance (g_s_), and transpiration (E) values were measured at solar noon (14:00 h.; UTC—4) using a portable photosynthesis system (LI-6400, LICOR Inc., Lincoln, Nebraska, EE. UU) equipped with a 6 cm^2^ transparent chamber. Considering the vegetative vigor of this ecotype, the third leaf (mature and healthy) below the apex was always monitored, and five measurements were made per treatment (two plants per measurement). The airflow rate was set at 500 µmol s^−1^, and the CO_2_ concentration was kept constant at 400 µmol mol^−1^ using a CO_2_ injection system provided by the equipment manufacturer [48]. Before any measurement, the instrument was calibrated correctly, including zeroing and the use of drierite and soda lime. This procedure was strictly followed to avoid any temperature-induced zero shifts [49].

#### 4.2.5. Yield Response

The yield response was characterized by the harvest index (H_i_) and the crop yield (C_y_). For the H_i_, the grains dry matter is related to the hole plant dry matter, obtaining the percentage that the yield represents concerning the total dry matter produced [50], and it is calculated as follows:

H_i_ = (DM_g_/DM_T_)·100, (3)

where H_i_ is the harvest index (%), DM_g_ is the grain dry matter (g), and DM_T_ is the whole plant dry matter (g). The DMT was obtained by harvesting the plants and separating the structures (leaves, stems, and roots). First, the fresh weight of the three structures was measured, and then they were dried in an oven with forced circulation (MMM Group, VENTICELL 707, Munich, Germany) at 80 °C for 48 h to obtain the dry weight. To determine the dry matter of each structure, nine plants per treatment were used to evaluate these parameters.

For Cy, the cob harvest was carried out when the grain presented a milky white appearance [51] for fresh consumption. In the center of the row of each block treatment, a 5 m^2^ area was defined a manual ear harvest was done [52]. Fresh ear yield included the kernel rows number and the fresh weight of each ear.

### 4.3. Experimental Design

The experimental design of the trial was a completely randomized block. In this regard, three blocks were established perpendicularly to the terrain slope, with 43 m long rows. The experimental unit was composed of eight rows with a total of 200 plants, while the observation unit corresponded to the four central rows (Supplementary Figure S2). Two irrigation treatments (T1 and T2) were evaluated during V10 and VT phenological stages. The T1 was the control, scheduling the irrigation every seven days, following the practices of the local growers from the Lluta valley. For T2, there was no irrigation during 11 days, considered “drought stress”. Hence, six experimental units were established for this trial. The monitored plants were randomly selected considering that they were not damaged by pest insects and not close to the edges.

### 4.4. Statistical Analysis

For those variables monitored over time (repeated measurements), a factorial model was used considering the interaction between irrigation treatments, hours, and days (fixed effects). In addition, the blocks were incorporated as random effects in the following way:

Y_ijklm_ = μ + τ_i_ + δ_j_ + γ_k_ + (τδ)_ij_ + (τγ)_ik_ + (δγ)_jk_ + (τδγ)_ijk_ + b_l_ + ϵ_ijklm_, (4)

where Y_ijklm_ is the response at the i-th level of irrigation, and at the j-th day at k-th hour in the l-th block, µ is the overall mean of the response, τ_i_ is the fixed effects of the irrigation levels, δ_j_ is the fixed effects of the evaluation day, γ_k_ is the fixed effects of the evaluation hour, (τδ)_ij_ is the fixed effects of the interaction of irrigation levels and evaluation days, (τγ)_ik_ is the fixed effects of the interaction of irrigation levels and evaluation hours, (δγ)_jk_ is the fixed effects of the interaction of days and hours, (τδγ)_ijk_ is the fixed effects of the interaction of irrigation levels and evaluation days and hours, b_l_ is the change in the mean Y_ijklm_ level associated with the k-th block, and ϵ_ijklm_ is the error term associated with the Y_ijklm_ observation. 

On the other hand, for those variables that were evaluated only once, a model was used considering the irrigation treatments as a fixed effect and the blocks as a random effect:

Y_ijk_ = μ + τ_i_ + b_j_ + ϵ_ijk_, (5)

For all the analyses, compliance with the variance analysis’s assumptions was reviewed: independent errors, errors with normal distribution and µ = 0, and treatments with homoscedastic variances. The normal distribution of error was tested with the modified Shapiro–Wilks normality test [53], and the homoscedasticity of variances was evaluated with the Levene test [54]. If statistically significant differences were found through the analysis of variance (*p* < 0.05), the means were subjected to the LSD-Fisher multiple comparison test. Finally, the statistical analysis was performed in the Infostat software [55]. 

## 5. Conclusions

The physiology, growth, yield, and hourly water relations of ‘lluteño’ maize were evaluated during 22 days of induced drought conditions under high salt content and B excess in soil and irrigation water. The reduction of available water in the soil generated a greater hydraulic tension towards the leaf, inducing a stomatal modulation that allowed a significant net assimilation of CO_2_ even under drought stress. According to the obtained results, the obtained drought condition (ψ_leaf_ = −1.67 MPa) would not be important for ‘lluteño’ maize, since it maintained the same yield as in the normal irrigation condition. This would be explained in part by the furrow irrigation, which generates a great wet soil profile.

Finally, this observed response could be explained by genetics, because this species is recognized for its adaptation to conditions of high salinity and B toxicity. Considering this extreme environmental condition, it is an effect that should be studied in greater detail, mainly if it is necessary to generate irrigation thresholds for the maize crop.

## Figures and Tables

**Table 1:** Effects of the control (T1) and drought stress (T2) treatments on net assimilation (A), stomatal conductance (g_s_), and leaf transpiration (E) at the beginning and the end of the trial (days 1 and 11 after last irrigation) for the lluteño maize, during vegetative growth (V10) and flowering (VT) phenological stages.

	A (µmol CO_2_ m^−2^ s^−1^)				g_s_ (mol H_2_O m^−2^ s^−1^)				E (mmol H_2_O m^−2^ s^−1^)			
	V10		VT		V10		VT		V10		VT	
Irrigation												
T1	36.6		32.2		0.12		0.17		2.63		4.58	
T2	34.8		24.4		0.09		0.13		2.10		3.82	
Time												
1	38.2		30.3		0.13 a		0.16		1.93 b		4.17	
11	33.2		26.4		0.08 b		0.14		2.80 a		4.23	
Irrigation × Time	1	11	1	11	1	11	1	11	1	11	1	11
T1	32.1 b	41.1 a	31.7 a	32.7 a	0.15	0.10	0.17 a	0.17 a	2.09	3.17	4.11 b	5.05 a
T2	34.4 b	35.2 b	28.8 a	20.1 b	0.11	0.07	0.16 a	0.10 b	1.76	2.43	4.23 b	3.40 c
Significances												
Irrigation	0.49		0.03		0.20		0.09		0.18		0.08	
Time	0.02		0.02		0.02		0.03		<0.01		0.75	
Irrigation × Time	0.05		<0.01		0.83		0.02		0.37		<0.01	

For effects of single factors, values followed by the same letter in rows are not significantly different (LSD Fisher *p* > 0.05). For effects of interactions, values followed by the same letter in rows and columns are not significantly different (LSD Fisher *p* > 0.05).

**Table 2:** Yield and harvest index (%) for the irrigation treatments (T1 = Control; T2 = Drought).

Treatment	Yield (kg ha^−1^)		Harvest Index (%)	
	Mean	Standard Deviation	Mean	Standard Deviation
T1	5350	650	20.0 a	4.0
T2	5337	489	13.0 b	2.0

Values followed by the same letter in rows are not significantly different (LSD Fisher *p* > 0.05).

**Table 3:** Chemical characterization of soil and water at the study site (Rosario sector, Lluta valley). The values correspond to the median and standard deviation of electrical conductivity of the extract (ECe), pH, organic material (MO), charge concentration of calcium (Ca^2+^), magnesium (Mg^2+^), potassium (K^+^), sodium (Na^+^), chloride (Cl^−^), sulfate (SO_4_^2−^), boron (B), percentage of exchangeable sodium (PSI), and sodium adsorption ratio (SAR) in soils of the study site.

Parameter	Soil		Water	
	Median	Standard Deviation	Median	Standard Deviation
ECe (mS cm^−1^)	5.5	0.5	2.3	0.3
pH (dimensionless)	7.2	0.2	7.5	0.4
MO (%)	1.0	0.5	-	-
Ca^2+^ (meq L^−1^)	22.0	1.6	9.0	1.3
Mg^2+^ (meq L^−1^)	10.3	1.1	2.5	0.2
K^+^ (meq L^−1^)	2.1	0.2	0.9	0.1
Na^+^ (meq L^−1^)	17.8	3.6	11.0	1.2
Cl^−^ (meq L^−1^)	17.6	6.0	13.4	3.4
SO_4_^2−^ (meq L^−1^)	32.0	6.3	8.6	0.5
B (meq L^−1^)	21.1	4.1	15.3	1.7
PSI (%)	15.7	7.8	-	-
SAR (dimensionless)	-	-	4.6	0.5

## Data Availability

The datasets generated for this study are available on request to the corresponding author.

## Supplementary Materials

The following supporting information can be downloaded at: https://www.mdpi.com/article/10.3390/plants12071519/s1, Table S1: Mean meteorological conditions during the phenological stages of measurement of the water status of the lluteño maize crop at field capacity and drought stress, during vegetative growth (V10) and flowering (VT) phenological stages; Table S2. Mean soil water content at 0–40 cm depth for the control (T1) and drought stress (T2) treatments at 1, 7 and 11 days after last irrigation, during vegetative growth (V10) and flowering (VT) phenological stages; Figure S1: Geographical location of the experimental site, showing the Lluta valley extension with a false color composition with Sentinel 2 imagery; Figure S2: Diagram of the experimental design.
