# Supplementary material for: Response of Maize (Zea mays L.) to Drought under Salinity and Boron Stress in the Atacama Desert
Source: Plants (Basel). 2023 Mar 31;12(7):1519. doi: 10.3390/plants12071519 (PMC10097302; doi:10.3390/plants12071519)
Supplement: Supplementary file 1 [file plants-12-01519-s001.zip › plants-2188609-supplementary.pdf]

Table S1. Mean meteorological conditions during the phenological stages of measurement of the water status of the lluteño maize crop at field capacity and drought stress, during vegetative growth (V10) and flowering (VT) phenological stages.

Phenological stage	Day	Rainfall (mm)	Rs (MJ m^{-2} day^{-1})	Ta (°C)	HR (%)	u (m s^{-1})	VPD (kPa)
V10	1	0.0	29.6	17.6	71.0	0.87	0.63
	7	0.0	24.2	16.3	72.1	0.75	0.58
	11	0.0	30.5	17.3	67.1	0.92	0.75
VT	1	0.0	30.9	19.8	69.9	0.91	0.76
	7	0.0	30.2	20.0	63.9	0.94	0.90
	11	0.0	28.2	19.2	68.7	0.70	0.76

Rs = solar radiation; Ta = air temperature; HR = relative humidity; u = wind speed; VPD= vapor pressure deficit.

Table S2. Mean soil water content at 0 – 40 cm depth for the control (T1) and drought stress (T2) treatments at 1, 7 and 11 days after last irrigation, during vegetative growth (V10) and flowering (VT) phenological stages.

	Soil water content (cm^3 cm^{-3})					
		**V10**			**VT**	
Irrigation						
T1		35.8			41.7	
T2		32.0			34.6	
Time						
Day 1		37.9			40.6	
Day 7		33.6			38.8	
Day 11		30.2			35.0	
Irrigation x Time	Day 1	Day 7	Day 11	Day 1	Day 7	Day 11
T1	36.5 ab	36.5 ab	34.3 a	41.6 a	41.2 a	42.2 a
T2	39.3 a	30.8 c	26.0 d	39.5 ab	36.5 b	27.8 c
Significances						
Irrigation		< 0.01			< 0.01	
Time		< 0.01			< 0.01	
Irrigation x Time		< 0.01			< 0.01	

For effects of single factors, values followed by the same letter in rows are not significantly different (LSD Fisher $p > 0.05$). For effects of interactions, values followed by the same letter in rows and columns are not significantly different for every phenological stage (LSD Fisher $p > 0.05$)

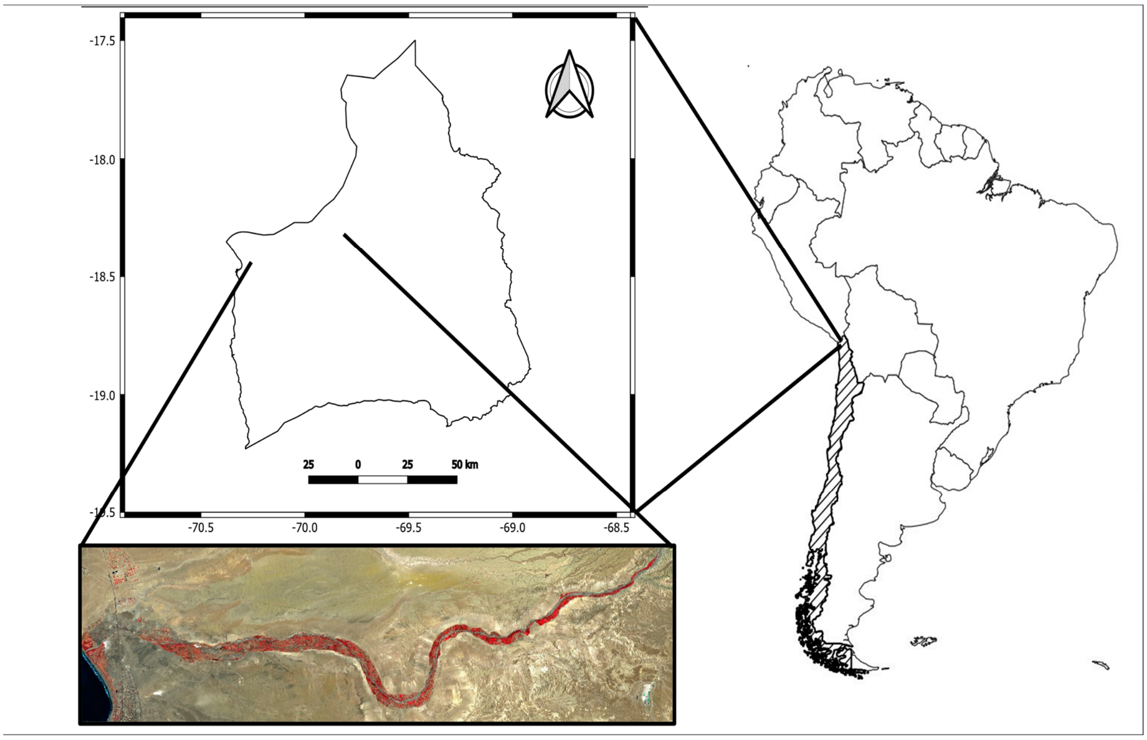

Figure S1. Geographical location of the experimental site, showing the Lluta valley extension with a false color composition with Sentinel 2 imagery.

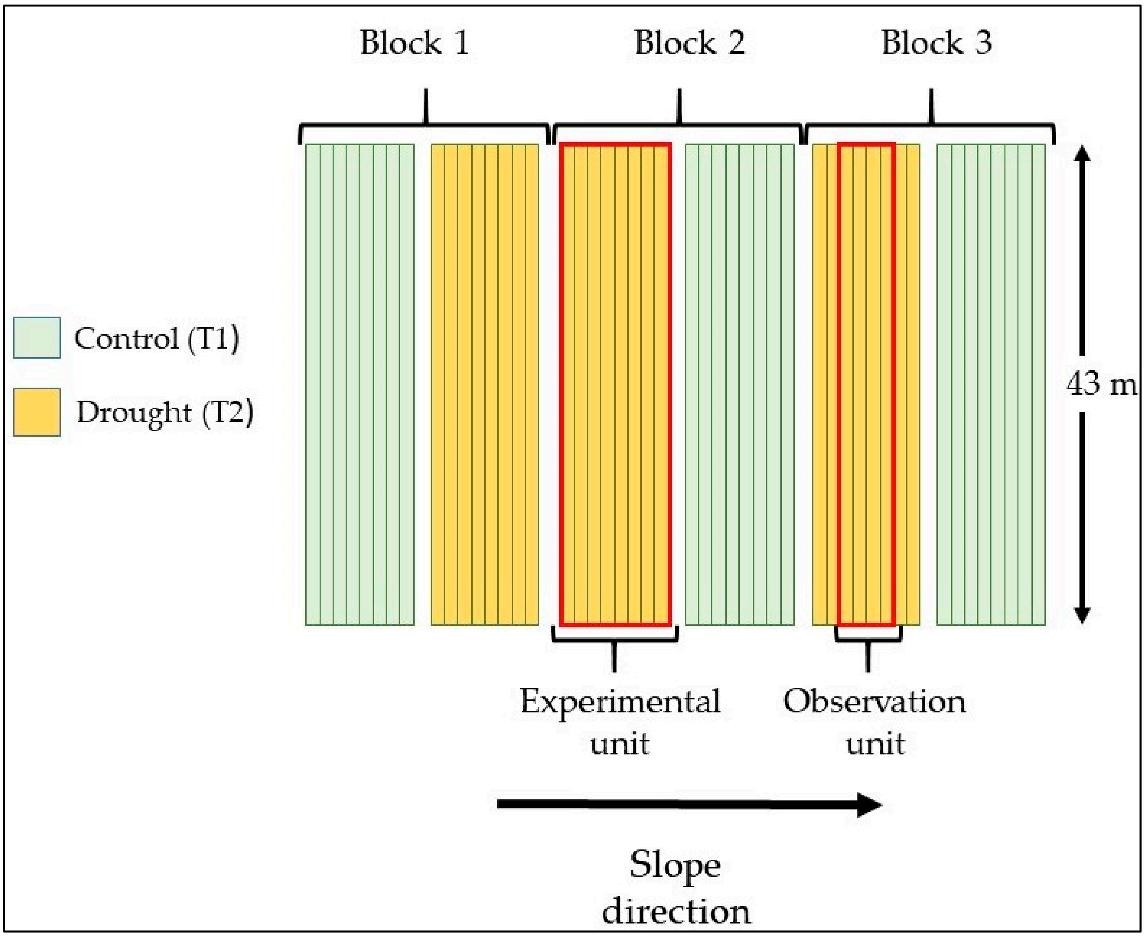

Figure S2. Diagram of the experimental design.
